# Transcriptomic Analysis Reveals the Effects of miR-122 Overexpression in the Liver of Qingyuan Partridge Chickens

**DOI:** 10.3390/ani14142132

**Published:** 2024-07-22

**Authors:** Xiaolu Luo, Jiahang Zhang, Jiancheng Guo, Wenjuan Zhao, Yinan Tian, Hai Xiang, Huimin Kang, Fei Ye, Siyu Chen, Hua Li, Zheng Ma

**Affiliations:** Guangdong Provincial Key Laboratory of Animal Molecular Design and Precise Breeding, School of Life Science and Engineering, Foshan University, Foshan 528225, China; bortingraf@outlook.com (X.L.); jhzhang0606@163.com (J.Z.); 17853636008@163.com (J.G.); zhaowj0508@163.com (W.Z.); evanrosy0122@163.com (Y.T.); xh@fosu.edu.cn (H.X.); huimin_kang@fosu.edu.cn (H.K.); yefei0831@fosu.edu.cn (F.Y.); chensiyu@fosu.edu.cn (S.C.); okhuali@fosu.edu.cn (H.L.)

**Keywords:** miR-122, chicken liver, transcriptomics, target-gene prediction

## Abstract

**Simple Summary:**

The liver plays a critical role in maintaining health and normal functions in chickens. This study focuses on understanding how a specific small molecule called microRNA-122, which is found in large amounts in the liver, influences these functions. To do this, we used a specially designed virus to increase the levels of microRNA-122 in the liver of young chickens. By analyzing the changes in gene activity, we discovered that higher levels of microRNA-122 impact several vital processes. These include fat metabolism, the aging of cells, cell adhesion, and cellular communication. Additionally, we identified eight specific genes that microRNA-122 may regulate. These findings provide new insights into how microRNA-122 controls important liver functions and can help improve the health and productivity of chickens.

**Abstract:**

The liver of chickens is essential for maintaining physiological activities and homeostasis. This study aims to investigate the specific function and molecular regulatory mechanism of microRNA-122 (miR-122), which is highly expressed in chicken liver. A lentivirus-mediated overexpression vector of miR-122 was constructed and used to infect 12-day-old female Qingyuan Partridge chickens. Transcriptome sequencing analysis was performed to identify differentially expressed genes in the liver. Overexpression of miR-122 resulted in 776 differentially expressed genes (DEGs). Enrichment analyses, including Gene Ontology (GO), Kyoto Encyclopedia of Genes and Genomes (KEGG), and Gene Set Enrichment Analysis (GSEA) revealed associations with lipid metabolism, cellular senescence, cell adhesion molecules, and the MAPK signaling pathway. Eight potential target genes of miR-122 (*ARHGAP32*, *CTSD*, *LBH*, *PLEKHB2*, *SEC14L1*, *SLC2A1*, *SLC6A14*, and *SP8*) were identified through miRNA target prediction platforms and literature integration. This study provides novel insights into the molecular regulatory mechanisms of miR-122 in chicken liver, highlighting its role in key biological processes and signaling pathways. These discoveries enhance our understanding of miR-122’s impact on chicken liver function and offer valuable information for improving chicken production performance and health.

## 1. Introduction

The liver is the largest accessory gland in the digestive system, primarily responsible for lipid metabolism including synthesis, digestion, absorption, decomposition, and transportation. In poultry, over 90% of de novo lipid synthesis occurs in the liver [1], unlike in mammals where fat production mainly takes place in adipose tissue [2,3,4,5]. This activity is particularly heightened during the egg-laying period due to the increased demand for lipid synthesis required for egg formation, underscoring the liver’s critical role [6]. In addition to lipid management, the liver plays critical roles in detoxification and immunity [7], underscoring its critical role in systemic regulation and health maintenance.

MicroRNAs (miRNAs or miRs) are small non-coding RNAs (~18–25 nucleotides long) ubiquitous in eukaryotes, regulating gene expression by pairing with the 3′-untranslated regions (3′UTRs) of target genes. MiRNAs are involved in various biological processes including growth, development, immune response, metabolism, and diseases [6,8,9,10,11,12,13,14,15,16,17]. Among them, microRNA-122 (miR-122) is a liver-specific miRNA [18,19,20], making up about 70% of total miRNA in the adult liver [21,22] and is highly conserved among chordates [19]. In addition, miR-122 has been confirmed to be involved in liver cell differentiation [23,24,25], liver development [26], lipid metabolism [27,28,29], and iron metabolism in hepatocytes. It is also required for liver polyploidization and maintaining liver homeostasis [29,30]. Although multiple experiments have proven that miR-122 is specifically expressed and highly abundant in chicken embryos and adult chicken livers [20,31,32], current research on the role of miR-122 in the liver mainly focuses on mammals. There are significant differences between birds and mammals in certain metabolic processes: for example, in mammals, lipid synthesis occurs mainly in adipose tissue, whereas in birds this process is almost entirely restricted to the liver. However, few studies have described the mechanism of action of miR-122 in bird liver. In poultry, relevant studies have verified only *VNN1* [33], *PKM2*, *TGF-β3*, *FABP5*, *ARCN1* [32] and *P4HA1* [34] as genes targeted by miR-122 in chicken liver, through which miR-122 mediates and regulates liver metabolism. Despite the identification of a few target genes of miR-122 in chicken liver, there has been limited progress since 2019. This gap highlights the urgency to explore miR-122’s regulatory mechanisms in bird liver metabolism, which could reveal new insights into its multifunctional roles and evolutionary conservation.

This study aims to investigate the function of miR-122 in the liver of Qingyuan Partridge chickens, a renowned variety of the yellow-feathered chickens which constitute 32% of the chicken meat production in Southern China [35,36,37,38,39]. Selected for their high nutritional value and distinct flavor, these chickens represent an important livestock product in the Chinese poultry market. Utilizing RNA-seq technology, the study aims to analyze miR-122’s role, predict its target genes, and enhance the understanding of miR-122’s function in chicken liver. The findings could provide a valuable reference for miRNA research in poultry, and offer scientific bases for improving broiler and laying hen production efficiency.

## 2. Materials and Methods

### 2.1. Statement of Ethics

The trial was conducted at Foshan University, with the animal protocol undergoing review and approval by the Experimental Animal Welfare and Animal Experiment Ethics Review Committee of Foshan University (approval number: FOSU202103-28). This process was conducted in accordance with the guidelines established by the Animal Use Committee of the Ministry of Agriculture of China, Beijing, aimed at minimizing animal suffering throughout the study.

### 2.2. Establishment and Identification of Lentivirus-Mediated miR-122-5p Overexpression Vector

The construction of the lentivirus-mediated miR-122-5p overexpression vector involved several critical steps. Initially, the precursor sequence of miR-122-5p (5′-UGGAGUGUGACAAUGGUGUGUUUGU) was designed based on data retrieved from miRbase (https://www.mirbase.org/, accessed on 10 August 2021). It was then synthesized and cloned into a lentiviral expression vector. This vector also contained a green fluorescent protein (GFP) reporter gene, facilitating easy identification and tracking of the vector in later stages. Following standard molecular cloning techniques, the synthetic miR-122-5p precursor sequence was inserted into the lentiviral expression vector. The accuracy of this recombinant vector was confirmed through restriction enzyme analysis and DNA sequencing.

To ensure the reproducibility and reliability of the lentivirus production, we outsourced the cloning and lentivirus production to GenePharma Company (GenePharma, Shanghai, China). The details of the lentivirus preparation process were provided by GenePharma and are included in the Supplementary Materials.

This meticulous approach, certified by GenePharma’s standard protocols and international guidelines, ensured the successful creation of the lentivirus-mediated miR-122-5p overexpression vector. All experimental procedures adhered to international standards and guidelines, guaranteeing accuracy and reliability of the results.

### 2.3. Animals and Breeding Management

For this study, six 12-day-old, female Qingyuan Partridge chickens, each weighing approximately 70 ± 5 g, were selected. These chickens were randomly divided into two groups: a negative control for miR-122 group (NC) and an over-expression for miR-122 group (M), with three chickens in each group. Randomization was performed using SPSS version 27.0 (SPSS Inc., Chicago, IL, USA). Each chicken was assigned a random number generated using the RV.UNIFORM (0, 1) function in SPSS, and then sorted based on this random number and equally divided into the two groups. The animals were housed in cages and fed a basal diet. The experimental conditions included a controlled room temperature of 30–32 °C, air humidity maintained at 50–60%, and a light cycle of 20 h per day. Daily ventilation was performed for 30 min to ensure proper air circulation, and the chickens had ad libitum access to feed and water.

On the first day, each chicken in both groups was injected through a wing tip vein with 300 μL of lentivirus solution at a titer of 10^9^ transducing units (TU) per milliliter. The NC group was administered a negative control lentivirus specifically designed as a non-targeting control for miR-122 (referred to as NC-miR-122), while the M group received a miR-122-5p mimic lentivirus (OE-miR-122), designed to overexpress miR-122-5p.

After 7 days, all chickens were euthanized, and samples were collected from the lower left portion of the liver of each chicken. The samples were placed in 2 mL cryopreservation tubes, immediately snap-frozen in liquid nitrogen, and then stored at −80 °C in an ultra-low temperature freezer for further analysis.

### 2.4. RNA Extraction, Real-Time Reverse Transcription PCR, and Deep Sequencing

Total RNA was isolated from three liver samples per group using RNA extract (Servicebio, Wuhan, China). Tissues were homogenized in 1 mL of Trizol reagent per 100 mg tissue, followed by centrifugation at 12,000× *g* for 10 min at 4 °C. The supernatant was mixed with 380 μL of chloroform, vortexed, and left at room temperature for 3 min. After centrifugation at 12,000× *g* for 15 min at 4 °C, the aqueous phase was collected, combined with 550 μL of isopropanol, and precipitated at −20 °C for 15 min. The RNA pellet was washed with 75% ethanol, dried, and dissolved in 20 μL of DEPC-treated water. RNA integrity and concentration were measured using a Bioanalyzer 2100 (Agilent Technologies, Beijing, China).

Qualified RNA samples were used for qRT-PCR. Reverse transcription was performed using the SweScript RT I First Strand cDNA Synthesis Kit (G3330) (Servicebio). The specific steps and system are described in the Supplementary Materials and Table S1.

qRT-PCR was performed using SYBR Green I dye and a kit provided by Servicebio. U6 was used as the reference gene for normalization in the quantification of miR-122-5p, while GAPDH was used for normalization in the quantification of target genes. Expression levels were calculated using the 2^−ΔΔCt^ method. The total reaction volume was 20 μL, consisting of 40 cycles of pre-denaturation at 95 °C for 30 s, denaturation at 95 °C for 15 s, and annealing/extension at the specific temperature for each primer pair for 30 s. Dissociation curve analyses showed a single peak, indicating good primer specificity. All qRT-PCR experiments were repeated three times for reliability. The sequences of the primers used are detailed in Table 1.

Following the identification of potential target genes, primers for qRT-PCR were designed using tools from the National Center for Biotechnology Information (NCBI). The primers were synthesized by Sangon Biotech (Shanghai) Co., Ltd., Shanghai, China.

Once the model was successfully established, deep sequencing analysis could be performed. Deep sequencing was performed by Beijing Biomarker Biotechnology Co., Ltd. (Beijing, China) using the Illumina platform.

### 2.5. Quality Control

We stringently controlled the quality of the raw data by removing reads containing adapter sequences and those of low quality. Specifically, this included discarding reads with more than 10% undetermined bases (N) and reads where more than 50% of the bases had a quality score (Q) of 10 or less, to obtain high-quality clean data. The reads, after quality control cleaning, were aligned to the reference genome using HISAT2 [40] with default parameters, and only alignments with a minimum mapping quality score of 20 were retained. The aligned files were then processed with SAMtools [41], where reads with a mapping quality score lower than 20 were filtered out. The results were then sorted and indexed. Gene expression levels were quantified using featureCounts [42], with reads assigned to the corresponding genes based on the reference annotation. Ultimately, we successfully identified 13,005 expressed genes, determining their expression levels by the number of uniquely mapped Reads. Subsequent analyses, including differential expression analysis and functional enrichment analysis, were performed.

### 2.6. Statistical Analysis of Differentially Expressed Genes and Functional Enrichment Analysis

The obtained data were used to estimate gene expression and identify differentially expressed genes (DEGs). Differential expression analysis between sample groups was performed using DESeq2, yielding a set of DEGs between the two groups. During the detection of differential expression, a Fold Change (FC) ≥ 1.5 and *p*-value < 0.05 were used as the criteria for filtering. The Fold Change represents the ratio of expression levels between two groups of samples.

Gene Ontology (GO) offers a standardized framework to describe gene and protein roles across organisms, covering Biological Process, Molecular Function, and Cellular Component domains. GO enrichment analysis identifies significantly enriched biological pathways under specific treatments. Similarly, the Kyoto Encyclopedia of Genes and Genomes (KEGG) database helps understand gene functions and biological system networks. Post-identification of differentially expressed genes (DEGs) performing GO and KEGG enrichment analyses is crucial. GO terms and KEGG pathways with *p* < 0.05 were considered significantly enriched, indicating important roles under different treatment conditions.

### 2.7. Gene Set Enrichment Analysis (GSEA)

Utilizing high-quality data from quality control after deep sequencing, Gene Set Enrichment Analysis (GSEA) was conducted using the GO and KEGG databases. Gene sets with *p* < 0.05 and FDR < 0.05 were identified. Significantly enriched pathways were defined based on the following criteria: an absolute Normalized Enrichment Score (|NES|) > 1, a Nominal *p*-value < 0.05, and an FDR < 0.25. Parts of pathways meeting these criteria were then selected for constructing Enrichment Score (ES) plots. We performed GSEA using clusterProfiler tools on the platform BMKCloud (https://www.biocloud.net/, accessed on 25 December 2021).

### 2.8. Predicting the Target Genes of miR-122-5p

To identify the functional target genes of miR-122-5p in chicken liver, we utilized the miRNA target-gene prediction platforms TargetScan 8.0 (https://www.targetscan.org/vert_80/, accessed on 10 May 2023) and miRDB (https://mirdb.org/, accessed on 22 May 2023). For TargetScan, we considered genes with a cumulative weighted context++ score below −0.2 as potential targets. In miRDB, we selected genes with a target score above 50. These thresholds were provided by the respective platforms, chosen based on their stringency in identifying high-confidence targets.

### 2.9. Statistical Analysis

Data were subjected to statistical analysis using the *t*-test, performed with SPSS version 27.0 (SPSS Inc., Chicago, IL, USA). The results were expressed as means ± standard error (SE) to accurately reflect both the central tendency and the variability of the data, as using the standard error was not appropriate with only three replicates. GraphPad Prism version 8.0 (GraphPad Software, Boston, MA, USA) was utilized to analyze and visualize the data after qRT-PCR.

## 3. Results

### 3.1. Establishment of an Overexpression Animal Model for miR-122

**Figure 1:**
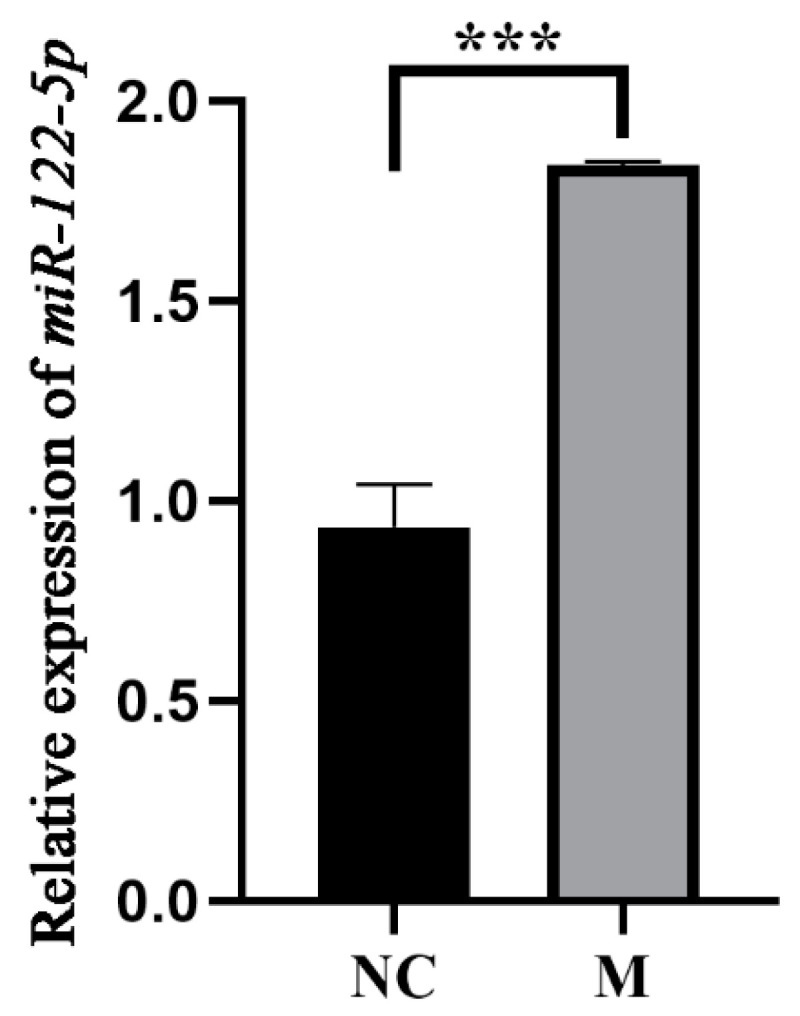
qRT-PCR measurement of miR-122-5p expression in both groups of samples. Using *t*-test, *** indicates *p* < 0.001. qRT-PCR analysis showed that miR-122 levels in the liver samples of the M group were significantly higher than in the NC group (*p* < 0.001). Values are means and standard error of the means, *n* = 3 per treatment. This confirms the successful creation of a miR-122 overexpression model in chicken liver through OE-miR-122 infection.

As illustrated in Figure 1, the expression levels of miR-122 in the liver samples of the M group were significantly higher than those in the NC group, as determined by qRT-PCR analysis (*p* < 0.001). This indicates the successful establishment of a chicken liver overexpression model for miR-122, achieved through the infection with OE-miR-122.

### 3.2. Transcriptome Data Quality Control Results

Following quality control measures for sequencing, key statistical data for each sample were acquired (Table 2). A comparison of clean reads and clean bases revealed variations in sequencing depth among the samples, providing critical insights for assessing sample quality and sequencing consistency. The GC content ranged between 48.75% and 50.22%, indicating a consistent genomic characteristic across the samples. Moreover, the percentage of Q30 bases in all samples exceeded 93.38%, demonstrating the high accuracy of the sequencing data. These high-quality data lay a solid foundation for subsequent analyses and ensure the reliability of the analysis results.

### 3.3. Identification of Differentially Expressed Genes

**Figure 2:**
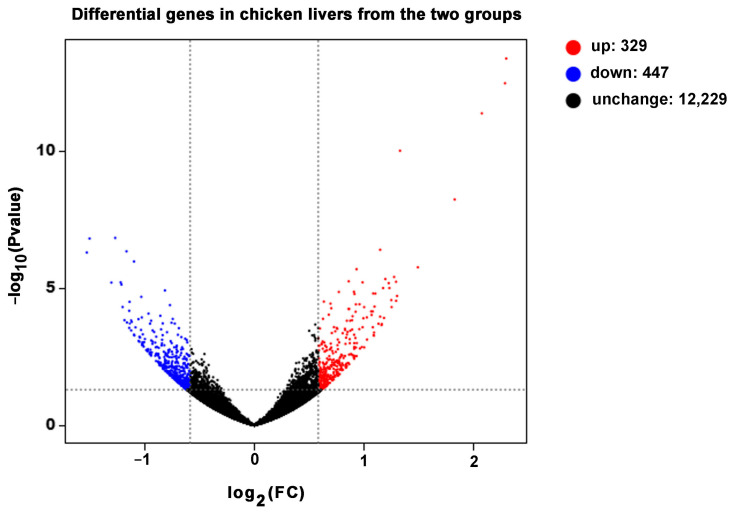
Number of differentially expressed genes. Through transcriptome sequencing and quality control, a total of 13,005 genes were identified. In the figure, red dots represent 329 up-regulated genes in the M group, while blue dots represent 447 down-regulated genes in the M group. The *x*-axis indicates log_2_(FC), where FC stands for Fold Change, and the *y*-axis represents −log_10_(*p* value). The dashed lines denote the thresholds (Fold Change ≥ 1.5, *p* < 0.05), plotted based on their log_2_(FC) and −log_10_(*p* value).

A total of 776 DEGs were identified by threshold (Fold Change ≥ 1.5, *p* < 0.05) screening (Figure 2, Table S3), and 329 genes were up-regulated and 447 genes were down-regulated in the M group compared with the NC group.

### 3.4. Functional Enrichment Analysis of DEGs: GO and KEGG Pathway Analysis

**Figure 3:**
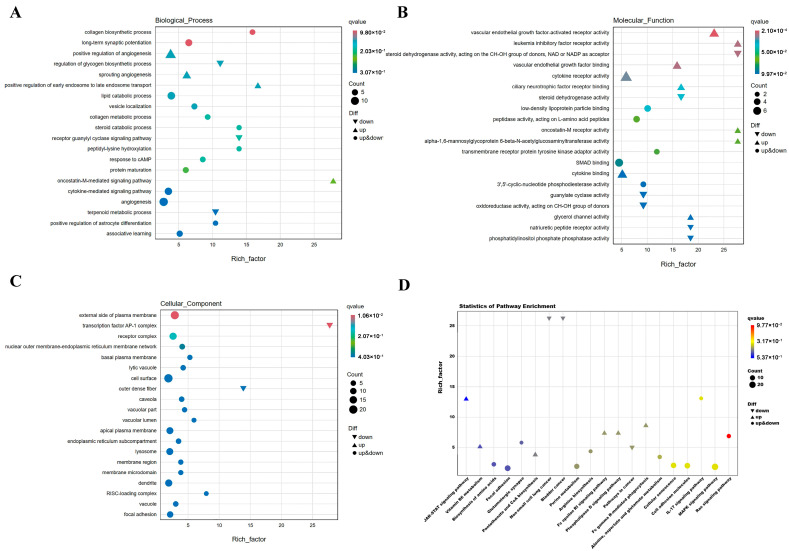
Functional enrichment analysis. (**A**) Go was used to analyze the Biological Process categories enriched by differentially expressed genes. (**B**) GO analysis of Molecular Function categories enriched for differentially expressed genes. (**C**) GO analysis of Cellular Component categories enriched by differentially expressed genes. (**D**) KEGG analysis of pathways enriched by differentially expressed genes. A total of 776 DEGs were subjected to GO and KEGG analysis. In (**A**–**C**), the *y*-axis represents the names of the gene-enriched GO categories, and the *x*-axis represents the Rich factor. The size of the dots corresponds to the count number, as indicated by the legend. Categories that comprise entirely up-regulated or down-regulated genes are marked with triangles, as shown in the legend. For (**D**), the axes are reversed compared to (**A**–**C**), with the *x*-axis representing KEGG pathway names and the *y*-axis indicating the Rich factor.

The functional enrichment analysis was performed on the identified 776 DEGs using both GO and KEGG pathway analyses, with the results depicted in Figure 3. As shown in Figure 3A, the DEGs were significantly enriched in GO biological process categories such as positive regulation of angiogenesis (GO:0045766), angiogenesis (GO:0001525), cytokine-mediated signaling pathway (GO:0019221), positive regulation of early endosome to late endosome transport (GO:2000643), and vascular sprouting (GO:0002040). Additionally, enrichment was observed in lipid catabolic process (GO:0016042) and steroid catabolic process (GO:0006706). Notably, genes enriched in the regulation of glycogen biosynthetic process (GO:0005979), receptor guanylyl cyclase signaling pathway (GO:0007168), and terpenoid metabolic process (GO:0006721) were predominantly down-regulated.

According to Figure 3B, DEGs were significantly enriched in GO molecular function categories including cytokine receptor activity (GO:0004896), vascular endothelial growth factor binding (GO:0038085), leukemia inhibitory factor receptor activity (GO:0004293), vascular endothelial growth factor-activated receptor activity (GO:0005021), alpha-1,6-mannosylglycoprotein 6-beta-N-acetylglucosaminyltransferase activity (GO:0030144), and oncostatin-M receptor activity (GO:0004924). Genes showing down-regulation were enriched in steroid dehydrogenase activity, acting on the CH-OH group of donors, NAD or NADP as acceptor (GO:0033764), steroid dehydrogenase activity (GO:0016229), guanylate cyclase activity (GO:0004383), natriuretic peptide receptor activity (GO:0016941) and phosphatidylinositol phosphate phosphatase activity (GO:0052866).

As indicated in Figure 3C, DEGs were notably enriched in GO cellular component categories such as transcription factor AP-1 complex (GO:0035976), external side of plasma membrane (GO:0009897), cell surface (GO:0009986), outer dense fiber (GO:0001520), and RISC-loading complex (GO:0070578). Particularly, genes enriched in outer dense fiber (GO:0001520) exhibited significant down-regulation.

KEGG pathway enrichment analysis revealed that the differentially expressed genes (DEGs) were significantly enriched in several key signaling pathways, including the MAPK signaling pathway (ko04010), Ras signaling pathway (ko04104), cellular senescence (ko04218), cell adhesion molecules (CAMs) (ko04514), IL-17 signaling pathway (ko04657), Phospholipase D signaling pathway (ko04072), JAK-STAT signaling pathway (ko04630), and focal adhesion (ko04510). Notably, these DEGs exhibited a down-regulation trend in pathways associated with cancer, particularly in the pathways of cancer (ko05200), bladder cancer (ko05219), and non-small cell lung cancer (ko05223), as clearly depicted in Figure 3D.

### 3.5. Gene Set Enrichment Analysis of DEGs

To further explore the differential gene expression patterns under normal and overexpression states of miR-122-5p in chicken liver, this study employed GSEA. The analysis was conducted based on 16,961 annotated sets from the GO database and 3545 annotated sets from the KEGG database. In this analysis, 64 significantly enriched gene sets were screened (Table S4). As shown in Table S4, we found that gene sets enriched or predominantly active under the overexpression state of miR-122-5p were primarily associated with biological functions closely related to the overall regulation of the organism. These functions include angiogenesis, negative regulation of apoptosis process, ovarian follicle cell development, phospholipid transport, protein kinase binding, melanosome, and transmembrane transporter activity. Additionally, these gene sets were up-regulated in several signaling pathways, such as regulation of the actin cytoskeleton, endocytosis, vascular smooth muscle contraction, cell adhesion molecules, MAPK signaling pathway, glycerophospholipid metabolism, FoxO signaling pathway, cellular senescence, arachidonic acid metabolism, lysosome, autophagy—animal, and ECM-receptor interaction.

**Figure 4:**
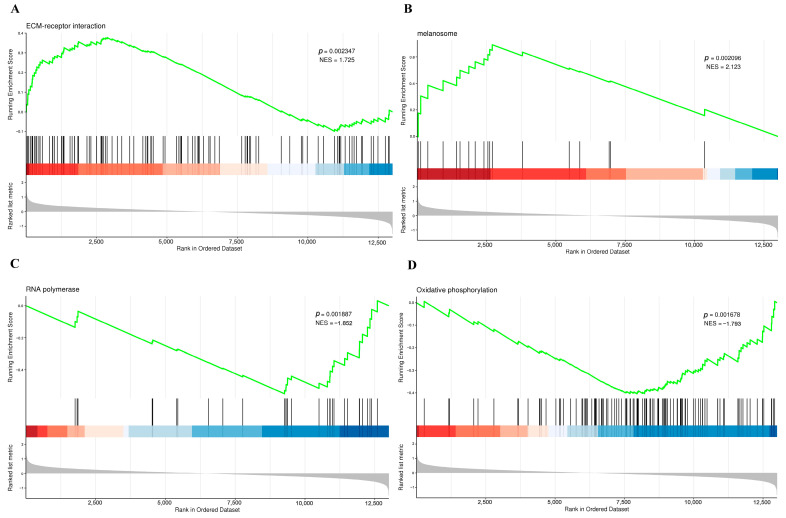
GSEA reveals significant enrichment of ECM interactions, melanosomes, RNA polymerase, and oxidative phosphorylation in the OE-miR-122 model. (**A**) The interaction between ECM and its receptors was significantly enriched in the experimental group (NES = 1.725, FDR < 0.05). (**B**) Significant enrichment of melanosome-related gene sets was demonstrated (NES = 2.123, FDR < 0.05). (**C**) Enrichment of RNA polymerase-related pathways (NES = −1.852, FDR < 0.05). (**D**) Significant enrichment of oxidative phosphorylation pathways (NES = −1.793, FDR < 0.05). The enrichment plot shows the rank of genes in the ordered dataset, with the green line indicating the running enrichment score and the color gradient below representing the ranked list metric, with red indicating higher ranks and blue indicating lower ranks. NES: normalized enrichment score; FDR: false discovery rate; green line: running enrichment score; bars: rank of genes in the ordered dataset.

Conversely, gene sets that were down-regulated under the overexpression state of miR-122-5p were found to be predominantly enriched in functions associated with translation, rRNA processing, and regulation of DNA metabolic process, among others. These include critical pathways like oxidative phosphorylation and ribosome function, suggesting a comprehensive impact on cellular metabolism and protein synthesis. Figure 4 shows the enrichment of biological processes partially discovered by GSEA, underscoring the potential impact of miR-122 in modulating various biological pathways within the chicken liver. GSEA revealed the potential role of different biological pathways in our study model, implicating the possible role of miR-122 when participating in chicken liver regulation.

### 3.6. Target-Gene Prediction Results

**Figure 5:**
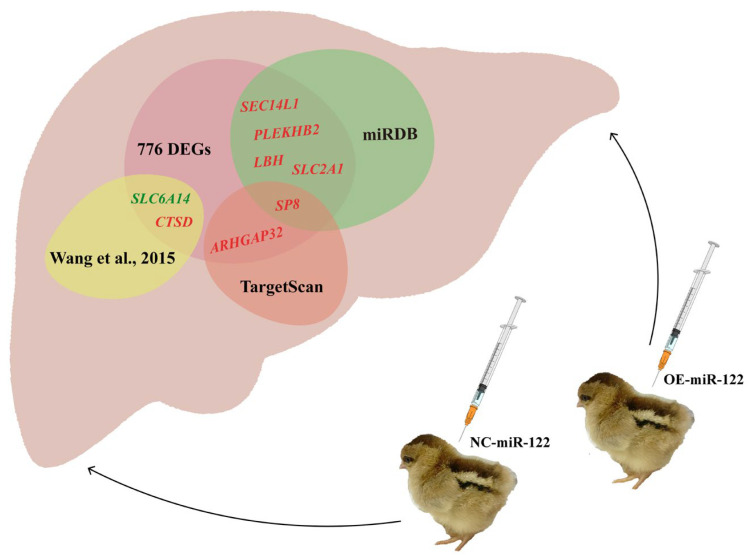
Prediction results of target genes. This figure shows the overlap of predicted target genes from different sources, including miRDB, TargetScan, and the study by Wang et al., 2015 [32]. Genes labelled in red indicate up-regulated expression in 776 DEGs, while green labels indicate down-regulated expression in 776 DEGs. miRDB: miRNA target prediction database; TargetScan: miRNA target prediction tool; Wang et al., 2015: Reference study for miR-122 target-gene prediction [32].

The results of miR-122-5p target-gene prediction are shown in Figure 5. The target genes of miR-122-5p in chicken/Gallus gallus were predicted by TargetScan 8.0 and miRDB, combined with DEGs in the NC group and the M group. *ARHGAP32*, *SP8*, *LBH*, *SLC2A1*, *SEC14L1*, and *PLEKHB2* were screened as possible target genes. In addition, the data in the article by Wang and colleagues [32] showed that, after miR-122 interference, the expression of the *SLC6A14* (ENSGALG00000005957) gene decreased (*p* < 0.05), and the expression of the *CTSD* (ENSGALG00000006613) gene increased (*p* < 0.05). *SLC6A14* and *CTSD* may be the target genes of miR-122 and participate in the physiological regulation of chicken liver.

### 3.7. Target Gene qRT-PCR Measurement Results

**Figure 6:**
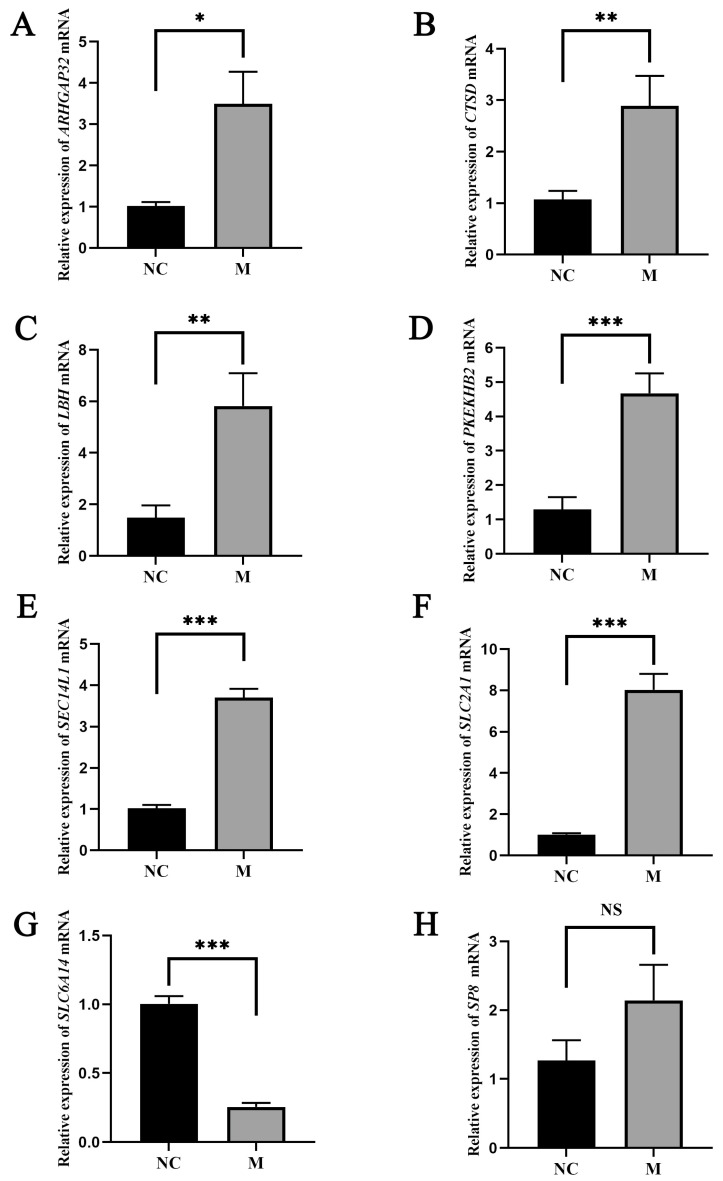
qRT-PCR measurement of target-gene expression levels in two groups of samples. (**A**–**H**) represent the mRNA levels of *ARHGAP32*, *CTSD*, *LBH*, *PLEKHB2*, *SEC14L1*, *SLC2A1*, *SLC6A14*, and *SP8*, respectively. In liver samples from the M group treated with overexpressed miR-122, *ARHGAP32* expression levels were significantly higher compared to the NC group (*p* < 0.05). *CTSD* and *LBH* showed highly significant increases (*p* < 0.01), while *PLEKHB2*, *SEC14L1*, and *SLC2A1* showed extremely significant increases (*p* < 0.001). Conversely, *SLC6A14* expression decreased significantly (*p* < 0.001). SP8 expression showed no significant difference compared to the NC group (*p* > 0.05). The qRT-PCR results for all eight genes were consistent with the transcriptome sequencing results. Values are means and standard error of the means, *n* = 3 per treatment. Using the *t*-test, *** indicates *p* < 0.001, ** indicates *p* < 0.01, * indicates *p* < 0.05, and NS indicates no significant difference.

Eight genes were selected as candidate target genes, for which primers were designed (Table 1), and qRT-PCR was performed. The qRT-PCR results are shown in Figure 6. In liver samples of the M group treated with overexpressed miR-122, the expression levels of *ARHGAP32* increased, showing significant differences compared to the NC group (*p* < 0.05); the expression of *CTSD* and *LBH* increased, showing highly significant differences compared to the NC group (*p* < 0.01); the expression levels of *PLEKHB2*, *SEC14L1*, and *SLC2A1* increased, showing extremely significant differences compared to the NC group (*p* < 0.001); the expression of *SLC6A14* decreased, showing significant differences compared to the NC group (*p* < 0.001). However, the expression of *SP8* in the M group showed no significant difference compared to the NC group (*p* > 0.05). The qRT-PCR results of all eight genes were consistent with the transcriptome sequencing results.

**Figure 7:**
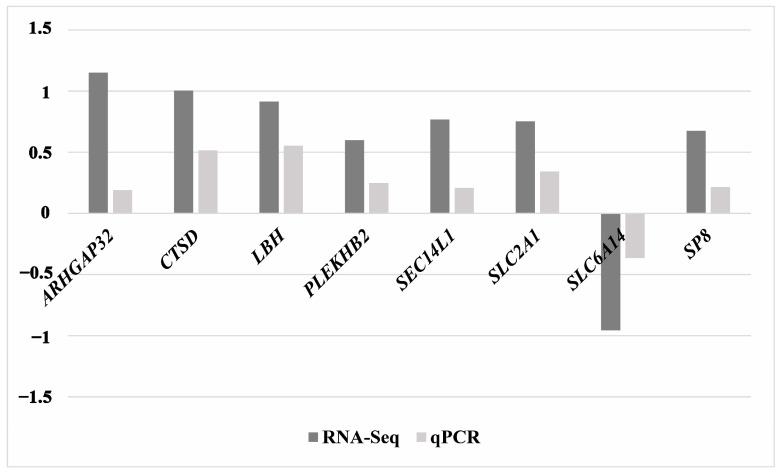
Comparison of gene expression levels in the NC vs. M groups using RNA-Seq and qPCR techniques. The vertical axis of this graph represents the log2(FC) in gene expression. A positive value indicates an up-regulation (increase in expression), and a negative value indicates a down-regulation (decrease in expression) of the gene. The bars represent different genes measured by two methods: RNA-Seq (shown in dark gray) and qPCR (shown in light gray). The qRT-PCR results for all eight genes were consistent with the transcriptome sequencing results.

To further explore the variations and magnitudes of gene expression levels between the M group and the NC group, as determined by qRT-PCR and transcriptome sequencing, Figure 7 presents a bar chart that illustrates these differences. The data shown are the log_2_(FC) of the expression levels, highlighting the relative changes calculated from both qRT-PCR and transcriptome sequencing results. This graphical representation highlights the consistency and magnitude of expression changes across the two techniques.

## 4. Discussion

miR-122 is well-known for its specific and highly abundant expression in chicken liver. The liver is crucial for growth, development, and maintaining homeostasis in many animals. In chickens, it is particularly important as the central organ for lipid synthesis [2,43,44,45]. This study aims to systematically elucidate the molecular regulatory mechanisms of miR-122 in the chicken liver, providing evidence and insights into its roles. Using RNA-Seq technology, our experiment identified 776 DEGs whose expression was altered due to the overexpression of miR-122 in chicken liver tissues. Further GO and KEGG analyses revealed that these DEGs are enriched in functions and biological pathways related to lipid metabolism (GO: 0016042 and GO: 0006706), glycogen synthesis regulation (GO: 0005979), and inflammatory response (ko04657). This provides clues about the specific high expression and systematic regulatory roles of miR-122 in the liver, contributing to a deeper understanding of the functions of miR-122 in the liver of chickens and birds in general. Additionally, GSEA uncovered significant associations of miR-122 with various biological processes and pathways, particularly those involved in cellular metabolism, growth regulation, and cellular senescence. This warrants further investigation to better comprehend the molecular regulation and functions of miR-122 in the chicken liver.

It is noteworthy that in our study, by utilizing the DEGs and combining the results of miRNA prediction platforms with previous research (Figure 5), we identified eight genes—*ARHGAP32*, *CTSD*, *LBH*, *PLEKHB2*, *SEC14L1*, *SLC2A1*, *SLC6A14*, and *SP8*—as potential target genes of miR-122. These genes, as potential targets of miR-122, warrant further investigation. However, a review of the literature revealed that these eight genes are scarcely mentioned in studies related to chicken liver, and their potential targeting relationship with miR-122 remains unexplored.

In alignment with existing interference experiment results, and exhibiting inverse expression levels, two genes, *SLC6A14* and *CTSD*, were identified. The former has been demonstrated as resulting in obesity, fatty liver, and metabolic syndrome in mice when the gene is knocked out under a high-fat diet [46]. Our study found that overexpression of miR-122 significantly reduced the expression level of *SLCA14* in chicken liver, indicating that *SLC6A14* is, indeed, regulated by miR-122, and that they have an inverse relationship. This suggests that *SLC6A14* plays a crucial role in mi-122-mediated regulation of liver lipid metabolism activities.

The latter, *CTSD*, has been identified as one of the major genes influencing yolk weight and egg weight. It also serves as a key enzyme in yolk formation and is critical for improving yolk traits through selective breeding [47,48]. Our results showed that overexpression of miR-122 significantly up-regulated *CTSD* expression in chicken liver, suggesting that *CTSD* is positively regulated by miR-122 and is involved in supporting the reproductive activities of laying hens.

The remaining six genes, which were identified as target genes with significantly increased expression levels in this study, are as follows: previous research has confirmed that *LBH* is a potential immunobiological marker for hepatocellular carcinoma [49], and it is highly conserved across different species. This finding implies that *LBH* could play a significant role in chicken liver, suggesting that miR-122 might regulate liver homeostasis by modulating *LBH* expression. *SEC14L1* has been shown to participate in the transfer of lipid-soluble vitamins, and plays a crucial role in lipid metabolism and signaling pathways [50]. Studies on *SLC2A1* indicate that its down-regulation in non-alcoholic fatty liver disease can promote lipid accumulation [51]. These findings suggest potential roles for *SEC14L1* and *SLC2A1* in chicken liver, further indicating that miR-122 may regulate hepatic lipid metabolism by modulating the expression of these two genes.

*ARHGAP32* is known to influence cell proliferation and differentiation [52], and it is also identified as a risk gene for neurodevelopmental disorders [53,54] and a potential therapeutic target for cancer treatment [55]. *PLEKHB2* has been recently associated with meat traits in sheep [56,57]. *SP8* is a critical factor that regulates *FGF8* expression and limb growth in vertebrate embryos, and its overexpression in chick embryos can promote *FGF8* expression [58]. Unfortunately, the specific roles and regulatory mechanisms of these genes in chicken liver remain largely unexplored.

In the introduction to this paper, we mentioned several previously identified miR-122 target genes, including *VNN1* [33], *PKM2*, *TGF-β3*, *FABP5*, *ARCN1* [32], and *P4HA1* [34]. However, in our transcriptome analysis, these validated genes did not exhibit significant differential expression. This discrepancy may be attributed to the limited sample size in our experiment, which constitutes a potential limitation. The small sample size can introduce variability that might skew the findings, masking real biological effects or creating false positives. These factors limit our ability to draw definitive conclusions about the regulatory networks of miR-122 in chicken liver, restricting the generalizability and statistical significance of the results.

Our study addresses a critical gap in the existing research by, for the first time, associating *ARHGAP32*, *CTSD*, *LBH*, *PLEKHB2*, *SEC14L1*, *SLC2A1*, *SLC6A14*, and *SP8* with the potential regulatory network of miR-122 in chicken liver. Although we observed the effects of miR-122 overexpression, its efficacy requires further validation. Additionally, the targeting relationships between miR-122 and its potential target genes were not validated in this study. Therefore, these findings require further experimental validation to elucidate the precise roles and mechanisms of these genes within the miR-122 regulatory network.

By addressing these limitations, future studies can enhance the reliability and applicability of the results, contributing to a more comprehensive understanding of miR-122 and its role in avian biology. Our research not only proposes new directions for investigation but also provides a solid foundation for future work. Subsequent studies should focus on increasing sample size and diversity to verify the biological significance of these potential target genes and to explore their roles in chicken-liver function.

## 5. Conclusions

In summary, this study investigates the role of miR-122 in chicken liver using transcriptomics and bioinformatics tools (target prediction tools, GO, and KEGG analysis). We identified eight key target genes—*ARHGAP32*, *CTSD*, *LBH*, *PLEKHB2*, *SEC14L1*, *SLC2A1*, *SLC6A14*, and *SP8*—linked to lipid metabolism, glycogen synthesis regulation, and inflammatory response. Unlike previously reported miR-122 targets (e.g., *VNN1*, *PKM2*, and *TGF-β3*), these genes did not show significant differential expression in this study, possibly due to our limited sample size. Additionally, we did not conduct targeted validation experiments, which is a limitation of this study.

Beyond the context of chicken liver, miR-122 has been widely studied in other species, particularly in human and mouse liver, where it plays crucial roles in liver development, disease, and metabolism. The insights gained from this study may have broader implications for understanding miR-122 function across different species and contexts.

Future research should not only aim to validate these findings with larger and more diverse sample sizes in chickens, but also explore the roles of miR-122 and its target genes in other tissues and species. This would enhance our understanding of the conserved and divergent functions of miR-122, thereby contributing to the generalizability and applicability of the miR-122 regulatory network findings across avian species and beyond.

## Figures and Tables

**Table 1:** Primer sequences for reference genes and target genes.

Gene	Direction	Sequence (5′-3′)	Tm (°C)	Product Size (bp)
gga-miR-122-5p	F	GATGCTCTGGAGTGTGACAATG	60	127
	R	TATGGTTGTTCACGACTCCTTCAC		
U6	F	CGCTTCGGCAGCACATATAC	60	148
	R	TTCACGAATTTGCGTGTCATC		
GAPDH	F	TCTTCACCACCATGGAGAAG	60	154
	R	CAGGACGCATTGCTGACAAT		
*ARHGAP32/ARHGAP33*	F	GACAACTGGAAGGACAGGGC	60	84
	R	ACCCCAGCAGCATCTCTTTC		
*CTSD*	F	ACAGCCATTGGTGCAAAACC	59	80
	R	GTGACAACAGGCAGAGACGA		
*LBH*	F	GCCGGGACTTCATGTCTGTG	61	133
	R	GAGAGCCCATCTTTACGGGG		
*PLEKHB2*	F	CGCGGCGGCTTTCGATTAAG	61	121
	R	CAAACGGCCATCAGACCACA		
*SEC14L1*	F	CGGTTGGAGCTGACCTTCAT	60	127
	R	TCAGCGGGCATGTAGGAAAC		
*SLC2A1*	F	CAAGATGACAGCTCGCCTGATG	61	150
	R	ATGGGCTCCTCATACGGTACA		
*SLC6A14*	F	GCCGTGCCTTTGGAATGTTT	59	94
	R	GGGAGGGTGCCATAACCAAA		
*SP8*	F	GGGCACTTTTGTGTGATGGC	60	137
	R	GACTGATAGCCCCGGTCAAG		

**Table 2:** Statistics of sequencing data.

Sample	Clean Reads	Clean Bases	GC Content	% ≥ Q30 ^1^
NC1	29,502,698	8,784,688,394	48.75%	94.05%
NC2	24,646,831	7,335,965,546	50.22%	93.38%
NC3	22,151,478	6,609,841,012	49.61%	93.81%
M1	23,481,002	7,011,105,812	49.18%	93.72%
M2	30,054,384	8,973,042,098	48.88%	93.52%
M3	20,590,795	6,146,105,976	49.15%	93.75%

^1^ The percentage of bases in the clean data with a quality score greater than or equal to 30.

## Data Availability

The original contributions presented in the study are included in the article/Supplementary Material; further inquiries can be directed to the corresponding author.

## Supplementary Materials

The following supporting information can be downloaded at: https://www.mdpi.com/article/10.3390/ani14142132/s1, Text S1: Details of the Establishment of Lentivirus-Mediated miR-122-5p Overexpression Vector. This section provides a comprehensive description of the methods used to establish the lentivirus-mediated miR-122-5p overexpression vector; Text S2: Details of the RNA Extraction and Real-Time Reverse Transcription PCR. This section details the protocols for RNA extraction, the procedures for real-time reverse transcription PCR, and the methodologies employed for deep sequencing; Table S1: Steps and System of Reverse Transcription Kit. This table outlines the detailed steps and components of the reverse transcription kit used in the study, providing information on the reagents and conditions for optimal reverse transcription; Table S2: The Composition and Nutrient Levels of the Basal Diet for Qingyuan Partridge Chickens. This table lists the ingredients and nutritional content of the basal diet formulated for Qingyuan Partridge chickens; Table S3: Differentially Expressed Gene. This table showed the 776 DEGs of this study; Table S4: Result of GSEA. This table presents the results of the functional enrichment analysis performed on the identified 776 DEGs using both GO and KEGG pathway analyses.
