# Supplementary material for: Transcriptomic Analysis Reveals the Effects of miR-122 Overexpression in the Liver of Qingyuan Partridge Chickens
Source: Animals (Basel). 2024 Jul 22;14(14):2132. doi: 10.3390/ani14142132 (PMC11274173; doi:10.3390/ani14142132)
Supplement: Supplementary file 1 [file animals-14-02132-s001.zip › animals-3116503-Supplementary materials.pdf]

Text S1. Details of the Establishment of Lentivirus-Mediated miR-122-5p Overexpression Vector

The process of preparing lentivirus is as follows:

A. Co-transfection of 293T Cells: First, package plasmids (PG-P1-VSVG, PG-P2-REV, PG-P3-RRE) and vector plasmid (Lentivirus vector) are co-transfected into 293T cells using a transfection reagent.

B. Virus Collection: After transfection, the 293T cells produce lentiviral particles. After a period of incubation, the culture medium containing lentiviral particles is collected.

C. Concentrating Virus: The collected culture medium containing lentiviral particles is then concentrated to increase the purity of the virus.

The above lentivirus vectors are provided by GenePharma Company (GenePharma, China).

The process of lentivirus packaging is as follows:

A. Cell Culture: Cultivate 293T cells in a 10 cm dish until they reach 80-90% confluence, and then seed the cells into a 15 cm dish.

B. Cell Washing: Discard the culture medium and wash the cells twice with 1 mL of D-Hank's solution.

C. Cell Digestion: Add 1 mL of Trypsin-EDTA solution to digest the cells.

D. Cell Collection: Remove the trypsin solution and add 2 mL of DMEM containing 10 % FBS to suspend the cells.

E. Cell Counting: Transfer the cell suspension to a centrifuge tube, centrifuge at 1,000 rpm for 5 min, discard the supernatant, and resuspend the cells in 1 mL of DMEM containing 10 % FBS. Perform cell counting.

F. Cell Seeding: Seed the cells into a 15 cm dish and add 20 mL of DMEM containing 10% FBS. Incubate the cells in a 37 °C, 5 % CO_2 incubator.

G. Transfection Preparation: In a sterile centrifuge tube, add 10 μg of shuttle plasmid containing the target sequence and packaging plasmids (pGag/Pol, pRev, pVSV-G), then add 1 mL of serum-free DMEM and mix well.

H. Transfection Mixture Preparation: In another sterile centrifuge tube, add 30 μL of transfection reagent, and then add 1 mL of serum-free DMEM. Mix thoroughly. Combine the solutions from the two centrifuge tubes and let stand at room temperature for 20 min.

I. Cell Transfection: Add the transfection mixture to the dish, gently swirl to mix, and incubate in a 37 °C, 5 % CO_2 incubator for 4-6 hours.

J. Medium Change: Remove the medium and add 20 mL of DMEM containing 10% FBS. Continue to incubate in the 37 °C, 5 % CO_2 incubator for 72 hours.

Through these steps, the shuttle plasmid containing the target sequence and the packaging plasmids (pGag/Pol, pRev, pVSV-G) are packaged into new lentiviral vectors.

Collection of Lentivirus

A. Supernatant Collection: Use a pipette to aspirate the supernatant from the culture dish and transfer it into a new 50 mL sterile, enzyme-free centrifuge tube. Pre-cool the centrifuge to 4 °C, set the centrifuge to 4,000 rpm, balance the tubes, and centrifuge for 4 min.

B. Filtration: After centrifugation, carefully aspirate the supernatant from the centrifuge tube using a pipette and transfer it into a 50 mL syringe. Attach a 0.45 μm filter to the syringe and filter the solution.

C. High-speed Centrifugation: Transfer the filtered solution into a new centrifuge tube, place it in a pre-cooled 4 °C centrifuge, balance the tubes, and centrifuge at 20,000 rpm for 2 h.

D. Concentrate Collection: Finally, remove the centrifuge tube and aliquot the concentrated solution into sterile, enzyme-free 2 mL microcentrifuge tubes. Store the processed aliquots at -80 °C.

Lentivirus Titer Assay

A. Cell Preparation: When 293T cells reach 80-90% confluence, discard the culture medium and wash the cells with 3 mL of D-Hank's solution (repeat this step once).

B. Trypsinization: Use a pipette to add 1 mL of Trypsin-EDTA solution to the cell culture dish, mix thoroughly, aspirate the excess trypsin solution, and incubate the dish at 37°C for 5 minutes.

C. Cell Suspension: Add 2 mL of DMEM containing 10% FBS to the culture dish and mix to suspend the cells.

D. Plating Cells: Seed the cell suspension into a 96-well plate at a concentration of 3 × 10^4 cells per well, and incubate for 24 hours.

E. Virus Dilution: Dilute 10 μL of the lentivirus stock solution in a serial dilution manner.

F. Infection: Discard the culture medium from the original 96-well plate and add 100 μL of the diluted viral solutions to each well using a pipette. Incubate for 24 hours.

G. Medium Change: After 24 hours, aspirate the viral solution from the wells, then add 100 μL of DMEM containing 10% FBS to each well. Continue to incubate in a cell culture incubator for 72 hours.

H. Titer Calculation: Observe the cells under a fluorescence microscope and count the fluorescent cells to calculate the viral titer.

Text S2. Details of the RNA Extraction and Real-Time Reverse Transcription PCR

Total RNA was isolated from three liver samples per group using RNA extract (Servicebio, Wuhan, China). First, the tissue was cut into small pieces and placed in a precooled homogenizer, 1 mL of Trizol reagent per 100 mg tissue, and the tissue was homogenized until there were no visible tissue pieces. Then, the samples were centrifuged at 12,000 g for 10 min at 4 °C to separate the tissue fragments, and the supernatant was transferred to a new centrifuge tube. To each 1 mL of RNA extract, 380 μL of chloroform was added, vortexed and mixed or the centrifuge tube was reversed vigorously for 15 s and left at room temperature for 3 min. After that, the upper colorless aqueous phase was carefully transferred to a new centrifuge tube by centrifugation at 12,000 g for 15 min at 4 °C, and approximately 450 to 500 μL could be collected. 550 μL of isopropanol was added to the collected aqueous phase, mixed gently upside down several times, and then precipitated by freezing at −20 °C for 15 min. The samples were centrifuged again at 12,000 g for 10 min at 4° C, and the white precipitate visible at the bottom was the total RNA. The supernatant was discarded, 1 mL of 75 % ethanol was added, mixed upside down, and centrifuged at 12,000 g for 5 min. The supernatant was discarded, briefly centrifuged at high speed (5,000 g, 3-5 s), and all liquid was carefully

aspirated with an aspirator. The centrifuge tube containing the RNA precipitate was left open for 3-5 min, and after the RNA was allowed to dry slightly, 20 μL of DEPC-treated water was added to fully dissolve the RNA. RNA integrity and concentration were measured using a Bioanalyzer 2100 system (Agilent Technologies, Beijing, China). All instruments and reagents were nuclease-free.

The qualified RNA samples were used for real-time quantitative reverse transcription polymerase chain reaction (qRT-PCR). The reverse transcription process was performed using the SweScript RT I First Strand cDNA Synthesis Kit (G3330) provided by Servicebio for first-strand cDNA synthesis. The specific steps and system are as follows (Table S1):

Table S1. Steps and system of reverse transcription kit.

Component	Volume
5×Reaction Buffera	4 μL
Oligo (dT)18 Primer (100)	1 μL
SweScript RT I Enzyme Mixb	1 μL
Total RNA	2 μL
Nuclease-Free Water	12 μL
Total	20 μL

The RNA concentration was 1000 ng/μL, and the above liquid was thoroughly mixed, followed by the following reaction conditions: 25 °C for 5 min, 50 °C for 30 min, and 85 °C for 5 s followed by -20 °C for further storage.

*a includes dNTP Mixture and Mg^{2+}.

*b includes RNase inhibitor.

qRT-PCR was performed using SYBR Green I dye and a kit provided by Servicebio. As a reference gene, U6 was chosen to normalize expression. Expression was calculated using the $2^{-\Delta\Delta Ct}$ method for relative quantification. The total volume of the reaction system was 20 μL. The specific procedure consisted of 40 cycles of predenaturation at 95 °C for 30 s, followed by denaturation at 95 °C for 15 s, followed by 30 s at the specific annealing/extension temperature for each primer pair. All dissociation curve analyses showed a single peak, indicating good primer specificity and suitability for qRT-PCR analysis. Table 2 lists the qRT-PCR primers for the target genes. Melting curve analysis was performed with default Settings. All qRT-PCR experiments were repeated three times to ensure the reliability of the data. Data were also analyzed using the $2^{-\Delta\Delta Ct}$ method.

Following the identification of potential target genes, we designed primers for quantitative PCR analysis. The primer design was facilitated by tools available through the National Center for Biotechnology Information (https://www.ncbi.nlm.nih.gov/), ensuring that the primers met specific criteria for melting temperature, GC content, and primer length to optimize assay specificity and efficiency. The designed primers were synthesized by Sangon Biotech (Shanghai) Co., Ltd. The sequences of these primers are detailed in Table 1.

Table S2. The composition and nutrient levels of the basal diet for Qingyuan Partridge chickens.

Ingredients	%	Calculated nutrient levels[1]	**%**
Corn	39.07	MJ（kcal/kg)	12.35

Wheat	25.00	CP	21.50
Soybean meal (43%CP)	22.00	EE	3.40
Peanut meal	3.00	CF	2.50
Corn gluten meal (58%CP)	5.00	Ca	0.90
Limestone	1.45	Total P	0.60
$CaHPO_4$	1.10	L-Lysine	1.38
L-lysine sulfate	0.64	DL-Met	0.61
DL-Met	0.32	L-Thr	0.82
NaCl	0.28		
L-Thr	0.19		
Lard oil	1.45		
Vitamin-mineral premix[2]	0.50		
Total	100.00		

[1]Values were calculated from data provided by China Nutrient Requirements for yellow-feathered broilers (2020).

[2]The premix provided the following per kg of diet: VA, 6,000 IU; VD_3, 2,000 IU; VE, 30 mg; VK_3, 2 mg; VB_1, 3 mg; VB_2, 5 mg; pantothenic acid, 800 mg; choline chloride 1,500 mg; nicotinic acid, 30 mg; pyridoxine, 3 mg; folic acid, 500 mg; biotin, 0.2 mg; VB_{12}, 1 mg; Fe, 100 mg; Cu, 8 mg; Mn, 100 mg; Zn, 100 mg; I, 0.42 mg; Se, 0.3 mg.
